# Splanchnic Vein Thrombosis in Myeloproliferative Neoplasms: Treatment Considerations and Unmet Needs

**DOI:** 10.3390/cancers15010011

**Published:** 2022-12-20

**Authors:** Angela Liu, Leonard Naymagon, Douglas Tremblay

**Affiliations:** Tisch Cancer Institute, Icahn School of Medicine at Mount Sinai, New York, NY 10029, USA

**Keywords:** splanchnic vein, thrombosis, myeloproliferative, polycythemia vera, essential thrombocythemia, myelofibrosis

## Abstract

**Simple Summary:**

Splanchnic vein thromboses (SVTs) are atypical clots associated with myeloproliferative neoplasms (MPNs). However, there are no well-established guidelines on how to treat them. The aim of this review is to explore treatment considerations of SVT in the setting of MPN (MPN-SVT). Anticoagulation is the cornerstone of therapy and cytoreductive therapy is recommended per MPN treatment guidelines. Endovascular intervention may also be considered in patients with occlusive or extensive clot burden in the acute setting to prevent or mitigate potential portal hypertensive complications. Beyond these general approaches, there are still gaps in our knowledge of how to treat MPN-SVT, including the optimal dose and timing of anticoagulation, role of endovascular interventions and novel agents, and management of patients with MPN-SVT without elevated counts. Future studies will be needed to bridge the gap of these unmet needs.

**Abstract:**

Patients who develop splanchnic vein thrombosis (SVT) in the setting of a myeloproliferative neoplasm (MPN) are at risk for complications including portal hypertension, bleeding, thrombosis, and death. Prompt multidisciplinary treatment is thus necessary to prevent long-term sequelae. However, optimal management strategies are not well established due to a paucity of data. In this review, we very briefly discuss the epidemiology, pathophysiology, and prognosis of MPN-SVT and then more comprehensively explore treatment considerations of MPN-SVT, including anticoagulation, endovascular/surgical intervention, and cytoreductive therapy. We will also highlight current gaps in our knowledge of MPN-SVT and conclude by suggesting future directions to optimize the treatment of MPN-SVT and improve outcomes.

## 1. Introduction

Thrombosis within the splanchnic veins (SVT) is a rare, atypical site of clot that is interestingly enriched in patients with Philadelphia-negative myeloproliferative neoplasms (MPNs)—polycythemia vera (PV), essential thrombocythemia (ET), and primary myelofibrosis (PMF) [1]. These MPNs are associated with mutations in *JAK2*, *CALR*, and *MPL*, all of which result in activation of the *JAK/STAT* pathway, leading to a proliferation of myeloid cells and elaboration of inflammatory cytokines [2]. PV is hematologically defined by an increased red blood cell mass, while ET is characterized by elevated platelets. PMF is associated with splenomegaly, bone marrow fibrosis, and can be present with either elevated blood counts or cytopenias. The major complications of MPNs include arterial and venous thrombosis (particularly with PV and ET), bleeding, and transformation to an aggressive form of acute myeloid leukemia termed MPN in blast phase (MPN-BP) [3,4].

SVTs include thromboses in the hepatic vein (also known as Budd–Chiari syndrome or BCS), portal vein (PVT), splenic vein, or mesenteric vein. Untreated or chronic SVTs can lead to intestinal ischemia as well as portal hypertension with manifestations including varices, splenomegaly, and liver failure [5]. SVTs are often provoked in the setting of cirrhosis, abdominal malignancy, and surgery. However, in the absence of these risk factors, MPNs are the leading cause of SVT [6,7].

The complications of SVT in the setting of MPN (MPN-SVT) span both disorders and include bleeding, thrombosis, sequelae of portal hypertension as well as MPN disease transformation [8,9]. Prompt and aggressive treatment is necessary to prevent long-term sequelae, but optimal management strategies are not well established due to a paucity of data involving this rare patient population. In recent years, there have been a growing number of studies on this clinical entity that can inform multi-disciplinary care that frequently includes hematology, hepatology and interventional radiology. In this review, we briefly discuss the pathophysiology and prognosis of MPN-SVT and then explore treatment considerations of MPN-SVT and the current gaps in our knowledge and care.

### 1.1. SVT as a Manifestation of MPNs

There are limited studies on the prevalence of SVT among MPN patients, but it is estimated that SVTs occur in 5–10% of patients with PV, 4–13% of patients with ET, and 0.6–1.0% of patients with PMF (Table 1) [10,11,12,13]. Development of an SVT in the setting of a *JAK2* mutation may portend later development of an MPN. In a meta-analysis, 21 (52.4%) out of 41 patients who harbor *JAK2V617F* without an MPN diagnosis at the time of SVT subsequently developed MPN during follow-up [14]. SVTs can also occur at the time of MPN diagnosis. In one large multicenter retrospective study, SVT and MPN were diagnosed concurrently (within 3 months of each other) in 240 out of 509 patients (47.1%) with a history of both SVT and MPN [15]. Lastly, patients with MPN may develop SVT later in their disease course. In a cohort study following 538 patients diagnosed with MPN under the age of 40, SVT occurred in 4.8% of patients during a median follow-up of 7 years [1]. SVT can occur prior to, at the time of, and after diagnosis of MPN and thus it is important to carry out interval reassessment of both disorders.

MPN-SVTs have certain features that distinguish them from more typical thromboses. Besides the atypical location, MPN-SVTs are often chronic at diagnosis, with studies demonstrating rates of chronic SVT in the 33–70% range [8,15,16], suggesting that the acute presentation of SVT is under-diagnosed. Chronic SVTs can lead to more complications including portal hypertension and are thus more morbid than chronic deep vein thromboses (DVTs) which can be associated with post-thrombotic syndrome [17,18]. Interestingly, traditional risk factors for thrombosis in MPNs including older age (>60) and prior history of thrombosis are not associated with MPN-SVT. In fact, multiple studies have found that MPN-SVTs are more prevalent in younger patients (median age <45), have a female predominance, and can occur in the setting of a low *JAK2V617F* allelic burden [15,16,19,20]. The reasons behind this interesting epidemiology are not well described, but may be related to other modulating risk factors such as hereditary thrombophilia and estrogen exposure.

### 1.2. Pathophysiology of MPN-SVT

The pathophysiology of this disorder is not entirely understood but is thought to involve interactions between activated MPN blood cells and the unique splanchnic environment, including hepatic endothelial cells. This is discussed further in detail in other narrative reviews by How et al. and Tremblay et al. [21,22]. According to Virchow’s triad, venous stasis, endothelial injury, and hypercoagulability are necessary in the development of VTE [23]. The splanchnic venous system may be susceptible to thrombosis because of slower blood flow and altered immunogenicity from the constant exposure of gut-derived antigens [21]. MPN patients have been found to have elevated levels of cell-free hemoglobin and nitric oxide in their blood, possibly leading to endothelial oxidative stress [24]. Additionally, MPN patients may be at risk for hypo-fibrinolysis, as one study demonstrated significantly elevated levels of plasminogen activator inhibitor type 1 (PAI-1), the inhibitor of tissue plasminogen activator (tPA), in the blood of MPN patients [25]. *JAK2*-activated blood cells create a procoagulatory, proinflammatory environment not only due to increased cell count leading to higher viscosity, but also due to changes in the cells themselves. In translational studies of MPN patients, it was found that platelets spontaneously aggregate [26], leukocytes secrete procoagulant molecules [27], and erythrocytes exhibit increased adherence to vascular endothelium [28]. Furthermore, *JAK2* is not only expressed in hematopoietic cells but is also likely present in endothelial cells of the liver and spleen, causing increased endothelial P-selectin expression and promotion of thrombosis [29,30]. However, the presence of *JAK2* endothelial cells in the liver has not been definitively demonstrated [31]. Given that the *JAK2* mutation is found in nearly all (80.3–86.6%) patients with MPN-SVT [7], this activating mutation is likely a major contributor in the pathogenesis of MPN-SVT. Altogether, the systemic procoagulatory state, activated myeloid cells, and endothelial cells (which may harbor the *JAK2* mutation) induce a prothrombotic milieu in an already vulnerable splanchnic system, subjecting MPN patients to a high risk of developing SVT.

### 1.3. Prognosis of MPN-SVT

There is a relative paucity of long-term studies of MPN-SVT patients. However, several studies have demonstrated an increased risk of morbidity and mortality, owing to both the hematologic complications of MPN compounded by the liver and intestinal dysfunction from SVT (Table 2) [8,9,15,16,32,33]. In a small study of 44 patients with MPN-SVT, 17 (39%) died during a median follow-up of 5.8 years, with a median age at death of 64 years old. Seven patients developed myelofibrosis and four progressed to MPN-BP, with eight patients (47%) ultimately dying from end-stage MPN. Three patients (18%) died from a new thrombotic event, with one developing intestinal ischemia related to extensive thrombosis of the superior mesenteric vein [8]. Although this appears to suggest that morbidity and mortality is largely related to MPN progression, a subsequent larger study questioned this finding. In a registry study with 3705 patients with PV or ET, Alvarez et al. demonstrated that after a median follow-up of 5.8 years, only 6% of patients progressed to myelofibrosis and none developed MPN-BP. However, the risk of death in patients with MPN-SVT patients was 2.47 times greater than in MPN patients without SVT. Contrary to the first study, this study demonstrated that death was more frequently from liver failure, major bleeding, and second malignancies rather than from MPN progression [9]. Notably, in the study by Alvarez et al., there was higher proportion of younger patients (median age 42 versus 48), patients on anticoagulation (AC, 67% versus 52%), and patients on cytoreductive therapy (77% vs. 68%) when compared to the population studied by Hoekstra et al. [8], which likely contributed to the difference in findings.

In addition to gastrointestinal (GI) complications and higher rates of death, MPN-SVT is also associated with an increased risk of thrombosis (intrasplanchnic and extrasplanchnic) and bleeding. One retrospective study demonstrated a recurrent thrombotic rate of 17.1% over 537 patient-years, or an incidence of 4.2 per 100 patient-years, with 45% occurring in the splanchnic veins and 55% occurring at other sites [33]. Given this high risk of recurrence, indefinite anticoagulation is often instituted as will be discussed below. However, the use of anticoagulation and antiplatelet therapy must be weighed against the risk of bleeding, which is also increased in this population due to the inherent characteristics of MPNs and the presence of esophageal varices in the setting of chronic SVT. In a large retrospective study of 519 MPN-SVT cases, 67% of patients presented with or developed gastroesophageal varices (GEVs) and 44.5% experienced one or more episodes of bleeding [15]. As described, the complications of MPN-SVT are potentially life-limiting and therefore it is critical to manage MPN-SVT at centers where multi-disciplinary care is employed.

## 2. Anticoagulation

### 2.1. Considerations Prior to Anticoagulation

Anticoagulation (AC) is the cornerstone therapy for SVT. AC averts clot progression, facilitates recanalization of thrombosed vessels, and decreases risk of future portal hypertension and recurrent thrombosis [35]. Retrospective data indicate that delaying AC may be associated with a higher risk of chronic thrombosis and portal hypertension [36]. For these reasons, AC should be initiated promptly. Nevertheless, the benefits of AC should be weighed against potential risks (many of which are unique to SVT), and efforts should be made to mitigate those risks where possible.

While AC should be started as early as possible, some patients have already developed portal hypertension by the time of diagnosis. This is particularly true of patients with hepatic vein thrombosis (BCS), which may have evidence of the stigmata of portal hypertension, including GEVs, at initial diagnosis. Portal hypertension is a less common finding in newly diagnosed acute portomesenteric vein thrombosis (PMVT), however, may develop over time (particularly if there is failure of recanalization), or may be present if PMVT is first diagnosed in the chronic phase. Ideally, patients with concern for portal hypertension should be screened for GEVs, and have their GEVS addressed prior to beginning AC, in order to minimize risk of variceal hemorrhage [37]. Portal hypertension may develop over time (particularly if recanalization is not achieved), or if mild at diagnosis may progress over time. Periodic screening for GEVs should therefore be considered if pursuing ongoing AC. Consideration may also be given to beta blockade to further reduce the risk of variceal bleeding complications. Among patients with MF, thrombocytopenia may be an additional consideration prior to anticoagulation, and if severe the relative risks and benefits of AC need to be carefully weighed.

Although prompt initiation of AC, as soon as deemed safe, is a critical first-line intervention, it may not be sufficient in many cases. In acute fulminant BCS, concurrent anticoagulation and endovascular intervention are often required to more rapidly decompress the portal circulation and avert liver failure. In many cases of fulminant BCS, decompression may be needed before AC can be safely started, as the tremendously high portal pressures magnify the risk of variceal hemorrhage [38]. Early endovascular intervention may also have a role in many instances of PMVT, as it may increase the likelihood of recanalization and decrease risk of future portal hypertension [35]. Such intervention should be strongly considered among patients harboring *JAK2V617F*, as successful recanalization may be unlikely, and portal hypertension commonly develops, if AC is pursued alone (as discussed further below) [39]. Thus, even when AC is started early, evaluation by an interventional radiologist with experience in the management of SVT, is advisable.

### 2.2. Choice of Anticoagulant

In the acute setting, if imminent endovascular intervention is being considered, or short-term bleeding risk is a concern, intravenous heparin is typically the AC of choice. Once outside this window (or if the above were never considerations), choices for long-term AC are manifold. Until recently vitamin-K antagonists (VKAs) and low-molecular-weight heparins (LMWHs) were the preferred ACs in the setting of SVT [40]. Direct oral anticoagulants (DOACs) were rarely used given that the clinical trials which established the use of DOACs in venous thromboembolisms (VTEs) included only patients with DVT of extremities and pulmonary emboli (PEs). In recent years, however, retrospective studies have observed that DOACs are safe and effective therapies in SVT, and that they compare favorably to VKAs and LMWHs in this setting [41,42]. Prospective data are now beginning to emerge to help support these findings. In a clinical trial conducted by Ageno et al. [43], 103 non-cirrhotic patients with acute SVT were treated with rivaroxaban and followed prospectively for 6 months. Among patients tested for the *JAK2V617F* mutation, 26% were positive, but interestingly, only 9% of the entire cohort had an MPN. Complete recanalization occurred in 47.3% of patients. Two patients had recurrent SVT (2.1%). Two patients had major bleeding events (2.1%) and there was one death due to a non-SVT-related cause (1.0%). The authors’ conclusions were that rivaroxaban appeared to be a reasonable alternative to standard AC in patients with non-cirrhotic SVT. Although most evaluations of DOACs in SVT have not been specific to MPN-SVT, their findings are likely generalizable, particularly given recent data regarding the comparable safety and efficacy of DOACs and VKAs in prophylaxis of VTE among MPN patients [44]. Despite the lack of definitive safety and efficacy data, we prefer DOACs for long-term AC among MPN-SVT patients due to their ease of use and patient convenience. This is in line with the recommendations of the International Society of Thrombosis and Haemostasis (ISTH) [45].

### 2.3. Duration of Anticoagulation

A diagnosis of MPN represents an ongoing risk factor for thrombosis. Recurrent events may occur within the splanchnic vasculature or at other sites, and risk may be highest among those patients harboring *JAK2V617F* [46]. Indefinite AC is therefore warranted following first SVT (or any other thrombotic event) in an MPN patient, although the risk–benefit assessment may prove dynamic as the disease evolves. Worsening portal hypertension and GEV burden, progression of thrombocytopenia, and development of bleeding complications, necessitate frequent reevaluation of the safety of ongoing AC. In some patients the risks of ongoing AC may eventually eclipse the benefits (particularly if recurrent and intractable bleeding arises). Patients receiving LMWH or DOAC may be trialed on prophylactic dosage should bleeding risk increase (with AC fully discontinued only if prophylactic dosage is not tolerated).

### 2.4. Efficacy of Anticoagulation and the Role of Endovascular and Surgical Intervention

As discussed above, AC prevents clot extension, promotes recanalization of occluded vessels, reduces the likelihood of future portal hypertension, and prevents recurrent thrombosis [35]. However, AC alone often proves unable to yield all these outcomes, particularly among patients with persistent pro-thrombotic risk factors such as MPNs. This may particularly be the case among patients harboring *JAK2V617F*. In a large retrospective cohort with acute non-cirrhotic PVT, those patients harboring *JAK2V617F* demonstrated a vessel-recanalization rate of only 16%, and nearly half developed portal hypertension during follow-up (outcomes were comparatively much better among patients with PVT due to other etiologies) [39].

These findings underscore that AC is likely not sufficient as monotherapy among many MPN-SVT patients, and that consideration should always be given to early endovascular intervention as an adjunct (particularly if clot burden is occlusive or extensive). Up-front vascular intervention is presently much better established in BCS (of all etiologies); however, some BCS patients with relatively benign symptoms are managed conservatively with AC alone [35]. MPN patients (particularly those with *JAK2V617F*) may be less likely to do well with such a conservative approach, and early endovascular intervention may be important in preventing or mitigating future portal hypertensive complications in this group (though further data are needed to help confirm this).

Liver transplantation may also be warranted for MPN patients with progressive BCS who fail conventional or endovascular interventions. One retrospective study demonstrated that MPN patients (*n* = 41) had similar long-term survival rates when compared to non-MPN patients (*n* = 37). After a mean 12.4 years of follow-up, no MPN patients progressed to overt MF or MPN-BP, even with standard immunosuppressive therapy. However, recurrent thrombosis and major bleeding occurred in 24% (7/29) of long-term MPN survivors. These findings reinforce the importance of conversations about the risk and benefit of certain interventions including surgery and AC in this population [47]. 

In the setting of chronic MPN-SVT, AC is unlikely to recanalize vessels or reverse or prevent portal hypertension (as the clot has already organized, adhered firmly to vessel walls, and caused local collateralization) [35]. However, AC still serves the function of preventing recurrent vascular events, and should therefore be started and continued even in the chronic phase.

## 3. Cytoreductive Therapy

In patients with PV and ET who have had a prior thrombosis, European LeukemiaNet (ELN) and National Comprehensive Cancer Network (NCCN) guidelines recommend cytoreductive therapy in order to mitigate the risk of subsequent thrombosis [48,49,50]. The most commonly used cytoreductive treatments in the front-line setting include hydroxyurea and recombinant interferon (pegylated interferon alfa-2a or ropeginterferon alfa-2b), for both PV and ET, and the *JAK1/JAK2* inhibitor ruxolitinib in PV patients who are hydroxyurea resistant or intolerant. Although there are substantial data to support this recommendation in patients with arterial or extra-hepatic venous thrombosis [51], the role of cytoreductive therapy in MPN-SVT is less clear. For instance, in a large European retrospective study of 1500 patients with MPN-related thrombosis, hydroxyurea treatment was not associated with a decrease in SVT recurrence (HR 0.81 95% CI 0.39–1.65). In contrast, this study showed that hydroxyurea was independently associated with a decreased rate of arterial and late venous thrombosis, suggesting that SVT may be unique among thrombotic events in the lack of efficacy for hydroxyurea in recurrence prevention [52]. An important statistical consideration of this study is that hydroxyurea exposure was treated as a categorical variable and the details of the timing, dose, and duration were not reported and presumably not incorporated into the analysis. In addition, although this study included 218 patients with MPN-SVT, it is unclear how many patients received hydroxyurea or other cytoreductive therapies. Therefore, this finding needs to be confirmed in larger, hopefully prospective studies. It is also not well established that cytoreductive therapy significantly improves portal hypertensive symptoms. We reported a single institution experience of 64 MPN-SVT patients and showed that there was no difference in time to development of gastroesophageal bleeding, ascites, hepatic encephalopathy, or recurrent SVT in patients treated with cytoreductive therapy versus those who were not [16]. Admittedly, the limitations of this small-sized retrospective study limit firm conclusion. However, it is fair to say that the role of cytoreductive therapy in reducing portal hypertensive symptoms and recurrence is not well established and further studies are needed.

Aside from these retrospective studies, other cytoreductive therapies have been explored prospectively. Ruxolitinib, which is approved for the treatment of myelofibrosis and PV, has been examined in a study of 21 MPN-SVT patients. These patients included 12 with myelofibrosis, 5 with PV and 4 with ET. Ruxolitinib was administered at a starting dose of 10 mg twice daily for PV, 25 mg twice daily for ET, and 15 mg twice daily for myelofibrosis for patients with platelet count 100–200 × 10^9^/L and 20 mg twice daily for patients with a platelet count of >200 × 10^9^/L. Nineteen patients (90%) were treated with concomitant VKA therapy. Ruxolitinib was well-tolerated in this patient population with a similar adverse event profile as seen in the non-SVT MPN population. Spleen volume reduction of at least 35% was observed in 29% of patients and there were significant reductions in MPN-related symptoms. However, there was minimal effect on esophageal varices and follow-up doppler US demonstrated stable, but not resolved, SVT and a stable resistive index of intraparenchymal splenic and hepatic artery [53]. In a separate study of 20 MPN-SVT patients with either ET or PV enrolled in the MPN Research Consortium 111 trial, pegylated interferon was evaluated in patients who were resistant or intolerant to hydroxyurea. Pegylated interferon was dosed at 45 µg weekly and up titrated each month by 45 µg based on tolerance and to induce a response. During the median follow-up on treatment of 114.2 weeks, there was one grade 3 esophageal bleeding event. One patient developed a DVT of the lower extremity one month after discontinuation of pegylated interferon. However, there were no recurrent SVTs noted during the follow-up period. Serial imaging was not protocol mandated so it is not clear if thrombosis resolution occurred [54].

In practice, we employ cytoreductive therapy in patients with MPN-SVT in patients with proliferative features, particularly those with PV or ET, despite the concrete evidence of benefit. This is in line with ELN and NCCN guidelines to provide cytoreduction to patients who have had a prior thrombotic event [48,49,50]. There is insufficient data to inform a preferred initial cytoreductive treatment. Therefore, we discuss the risks/benefits of both hydroxyurea or interferon therapy. In particular, in younger patients who have reproductive considerations, we typically employ recombinant interferon as it can be safely given during conception and pregnancy [55]. In addition, patients who suffer from constitutional symptoms or symptomatic splenomegaly, ruxolitinib is also considered, particularly in the case of MF. The role of cytoreductive therapy in patients without elevated blood counts is controversial. Particularly in patients with MPN-SVT, MPNs can be “masked” because of the dilutive effects of portal hypertension. We typically discuss the risks/benefits of this intervention with patients.

## 4. Unmet Needs

Despite decades since the association between SVT and MPNs was reported, there remains limited evidence to guide the management of MPN-SVT. Although anticoagulation is clearly beneficial to prevent thrombosis within and outside the splanchnic bed, there are limited investigations into the dose and duration of therapy. For instance, while DOACs appear to be safe and effective in patients with MPN-related thrombosis [44], it remains unclear if the de-escalation of dose is safe and effective as has been shown in other VTE [56,57]. In addition, the added utility of antiplatelet agents has not been specifically explored in this population of MPN patients who are typically on AC therapy. Ideally these questions should be answered in a prospective manner in the form of rationally-designed therapeutic clinical trials. However, with the rarity of MPN-SVT, this may not be possible. Additional information from observational, prospective, ambispective, or large retrospective studies. Collaboration between institutions will be essential in these efforts.

As previously discussed, recanalization rarely occurs in *JAK2*-mutated SVT patients despite AC and cytoreductive therapy. Therapeutic strategies, including endovascular interventions, that can be employed early in the disease course have only been peripherally studied. In addition, novel agents that can reduce the thrombotic burden and improve portal hypertensive symptoms are needed. For instance, given the likely expression of *JAK2* in the endothelial cells of the splanchnic vasculature and resultant increase in P-selectin expression [58], a P-selectin blocking antibody such as crizanlizumab may be effective in these patients. Further preclinical work is needed to identify potential targets to improve the outcomes of patients with MPN-SVT.

It is unknown if *JAK2V617F* screening alone is sufficient for the workup of MPN in patients with an unprovoked SVT. The *JAK2V617F* mutation is found in 80–87% of patients with MPN-SVT [7]. Testing for *CALR* and *MPL* genes may be helpful in diagnosing MPN (especially in *JAK2*-negative cases); however, *JAK2* testing should be prioritized as that mutation is the most prevalent. Interestingly, there can be a case made for more selective *CALR* testing. In one study [59], *CALR* testing in patients with spleen height ≥16 cm, platelet count >200 × 10^9^/L, and no *JAK2V617F* mutation, yielded a positive predictive value of 56% and negative predictive value of 99% for MPN. Using this strategy, the authors concluded that 96% of unnecessary *CALR* testing could be avoided. It is also unknown if next-generation sequencing (NGS) is necessary for the workup and management of MPN-SVT. NGS may be useful in identifying MPN not detected by conventional methods (e.g., *JAK2*-exon 12 mutations in triple-negative MPN-SVT [60,61]) and in prognostication, but ultimately, it is yet to be determined if the information gained from NGS significantly impacts management.

Another area of unmet need is understanding the clinical manifestation and management of SVT patients in the setting of an isolated *JAK2* mutation or without elevated blood counts, as is the case with many patients who have *JAK2* clonal hematopoiesis (CH) or an unclassifiable MPN (MPN-U). It is unknown whether *JAK2* CH increases the risk of SVT or if the development of SVT may be more closely related to the development of MPN. Patients with *JAK2* CH are at increased risk of DVT and PE [62,63] but there are no studies evaluating the prevalence or development of SVTs in this population. Current data suggest that the development of an SVT may be a high-risk feature for developing an MPN among *JAK2* CH patients. In a meta-analysis by Dentali et al. [14], 21 (52.4%) out of 41 patients with SVT who were found to have an isolated *JAK2V617F* mutation later developed an MPN. However, this is a small number of patients across multiple studies; therefore, larger prospective studies will be needed to validate this finding. Diagnostically, elevated counts may not be present in all MPN-SVT patients even if they have a proliferative MPN given hypersplenism causing hemodilution and GI bleeding. Therefore, it may be possible that many patients with normal counts may have an expanded red cell mass that can only be detected by isotopic measurement [9,64,65]. However, the prevalence of an increased red cell mass in MPN-SVT patients with normal counts has not been explicitly explored. As previously discussed, the impact of cytoreductive therapy has not been studied specifically in this population of MPN-SVT patients with normal blood counts, and it is not known at which platelet or hemoglobin/hematocrit thresholds we should begin cytoreductive therapy. Within the realm of cytoreductive therapy, it remains unclear if there are blood counts that should be targeted among patients with proliferative MPN-SVT. Previous work has questioned the validity of tight hematologic control on outcomes in PV and ET [66,67,68]; however, it is not clear if there are important WBCs, hematocrit, and platelet targets in patients with MPN-SVT.

## 5. Conclusions

**Figure 1:**
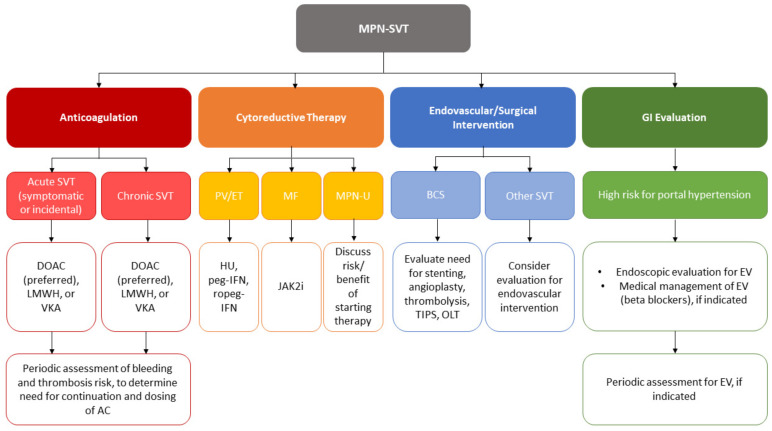
Management strategies for MPN-SVT. Multifaceted treatment of MPN-SVT is required including consideration for anticoagulation, cytoreductive therapy, endovascular/surgical intervention, and close coordination with hepatology. AC, anticoagulation; BCS, Budd–Chiari syndrome; DOAC, direct oral anticoagulant; GI, gastroenterology; HU, hydroxyurea; ET, essential thrombocythemia; EV, esophageal varices; *JAK2i*, *JAK2* inhibitor; LMWH, low molecular weight heparin; MF, myelofibrosis; MPN, myeloproliferative neoplasm; MPN-U, MPN-unclassifiable; OLT, orthotopic liver transplant; peg-IFN, pegylated interferon; PV, polycythemia vera; ropeg-IFN, ropegylated interferon; SVT, splanchnic vein thrombosis; TIPS, transjugular intrahepatic portosystemic shunt; VKA, vitamin K antagonist (warfarin).

MPN-SVT represents an interesting and unique clinical challenge that has gained a growing interest in recent years. Given the rarity of the disease, the broad range of presentations (acute versus chronic SVT, MPN-type), and dynamic course of complications that can arise (portal hypertension, bleeding, thrombosis, MPN progression, death), there are no well-established guidelines on management. A summary of the proposed management strategies can be found in Figure 1. Anticoagulation remains the mainstay of treatment to prevent clot extension but endovascular interventions may be needed to increase the likelihood of recanalization. Liver transplantation can be a potential option for patients who are not candidates for other therapies. If the SVT is chronic, gastroenterology involvement is needed to manage the portal hypertensive sequelae. Cytoreductive therapy is often utilized per MPN treatment guidelines; however, its impact on reducing the risk of MPN-SVT or progression of complications of MPN-SVT is unclear. Current gaps in our knowledge of MPN-SVT include the optimal dose and timing of anticoagulation, role of endovascular interventions and novel agents, and management of patients with MPN-U. We recommend the use of DOACs as they seem to be safe and effective in patients with MPN; however, more definitive data will be needed on their use in MPN-associated thrombosis. More studies will be needed to further optimize the treatment of MPN-SVT to reduce morbidity and mortality.

## Figures and Tables

**Table 1:** Prevalence rates of SVT among MPNs, prevalence rates of MPN among non-cirrhotic, non-local malignancy-related SVTs.

MPN	Prevalence of SVT	References
PV	5.5–10.0%	14/140 with SVT (10%) [10]
		13/235 (5.5%) with BCS and PMVT [11]
ET	4.0–13.0%	3/23 (13%) with SVT [10]
		19/460 (4%) with SVT [12]
		24/259 (9.2%) with BCS and PMVT [11]
PMF	0.6–1.0%	1/106 (1%) with SVT [10]
		4/707 (0.6%) with SVT [13]
**SVT**	**Prevalence of MPN**	**References**
PVT	31.5%	188/615 (31.5%) with MPN [7]
	27.5% PV	
	26.2% ET	
	12.8% MF	
	17.7% MPN-U	
	24.0% solitary *JAK2V617F*+	
BCS	40.9%	180/440 (40.9%) with MPN [7]
	52.9% PV	
	24.6% ET	
	6.7% MF	
	17.0% MPN-U	
	6.5% solitary *JAK2V617F*+	

BCS, Budd–Chiari syndrome; ET, essential thrombocythemia; MF, myelofibrosis; MPN, myeloproliferative neoplasm; MPN-U, MPN-unclassifiable; PMF, primary myelofibrosis; PMVT, portal mesenteric vein thrombosis; PV, polycythemia vera; SVT, splanchnic vein thrombosis.

**Table 2:** Complications of MPN-SVT.

Complication	Findings from MPN-SVT Studies	References
Bleeding	Occurrence 20–39%	13/64 (20%) [16], 17/44 (39%) [8]
	Time to first bleeding event: 2.3 yr (0.5–5.1)	[8]
	Incidence 1.2–2.4 per 100 pt-yr	1.2 per 100 pt-yr [15], 2.0 per 100 pt-yr [34], 2.43 per 100 pt-yr [9]
	IRR 3.6, 95% CI 2.3–5.5, *p* < 0.001 Higher risk of bleeding compared to MPN pts w/o SVT	[9]
Recurrent thrombosis	Occurrence 27% (12/ 44) [25% intrasplanchnic VTE, 33% extrasplanchnic VTE, 42% arterial]	[8]
	Time to recurrent thrombosis: 7.5 yr (0–18)	[8]
	Incidence recurrent thrombosis 4.2 per 100 pt-yr	[34]
	Incidence VTE 2.55 per 100 pt-yr [75% cases intrasplanchnic, 25% cases extrasplanchnic]	[9]
	Incidence SVT recurrence 1.6 per 100 pt-yr	[15]
	Incidence extrasplanchnic VTE 1.6 per 100 pt-yr	[15]
	Incidence arterial thrombosis 0.9–1.6 per 100 pt-yr	0.90 per 100 pt-yr [9], 1.6 per 100 pt-yr [15]
	Risk factors for recurrent thrombosis: BCS (HR 3.03), history of prior thrombosis (HR 3.62), splenomegaly (HR 2.66), leukocytosis (HR 2.8)	[34]
Gastrointestinal complications	EV occurrence 67% (307/458)	[15]
	Ascites occurrence 47% (30/64)	[16]
	Hepatic encephalopathy occurrence 9% (6/64)	[16]
	Splenomegaly occurrence: 56.3% PV-SVT, 46.9% ET-SVT	[15]
MPN progression	Occurrence 27% (12/44)	[8]
	Time to disease progression 9.7 yr (1–17)	[8]
	11% developed MF (7/80) or MPN-BP (2/80)	[32]
	6% progressed to MF (7/118), no cases of MPN-BP	[9]
	No difference in progression to MPN-BP compared to MPN pts w/o SVT Evolution to secondary MF was lower in PV-SVT (1.5 vs. 1.7, *p* = 0.034) compared to PV pts w/o SVT	[15]
	Risk factors for MPN progression or death: *JAK2V617F* allele burden ≥ 50% (OR 14.7), presence of chromatin/spliceosome/*TP53* mutations (OR 9.0)	[32]
Death	Occurrence 39% (17/44), most died from end-stage MPN Median age at death 64 yr (30–88) 1-yr OS, 98%; 5-yr OS 88%	Deaths: 8/17 (47%) from end-stage MPN, 3/17 (18%) from thrombosis [8]
	HR 2.47, 95% CI 1.5–4.01, *p* < 0.001, most died from hepatic disease, major bleeding, or second cancer	[9]
	Median OS similar in PV-SVT (24.9 vs. 23.8 yr, *p* = 0.76), shorter in ET-SVT (*p* < 0.001), longer in PMF-SVT (22.1 vs. 6.1 yr, *p* < 0.001) compared to controls (MPN pts w/o SVT)	[15]

BCS, Budd–Chiari syndrome; CI, confidence interval; ET, essential thrombocythemia; EV, esophageal varices; HR, hazard ratio; IRR, incidence rate ratio; MF, myelofibrosis; MPN, myeloproliferative neoplasm; MPN-BP, myeloproliferative neoplasm-blast phase; OR, odds ratio; OS, overall survival; pt-yr, patient-years; pts, patients; PV, polycythemia vera; SVT, splanchnic vein thrombosis; VTE, venous thromboembolism; yr, year(s).
